# The impact of airway management guided by Protection Motivation Theory on the prevention and prognosis of post-stroke pneumonia

**DOI:** 10.3389/fneur.2025.1579490

**Published:** 2025-09-18

**Authors:** Qiaoyun Liu, Liuqing Wang, Pan Xia, Lanjuan Li, Chen Zhao, Siqi Liu, Shanshan Xu

**Affiliations:** ^1^Department of Neurology, The Affiliated Gaochun Hospital of Jiangsu University, Jiangsu, China; ^2^Department of SCience and Education, The Affiliated Gaochun Hospital of Jiangsu University, Jiangsu, China

**Keywords:** airway management, Protection Motivation Theory, prevention, prognosis, post-stroke pneumonia

## Abstract

**Background:**

Stroke remains a leading cause of morbidity and mortality worldwide, with post-stroke pneumonia significantly impacting patient outcomes. Despite progress in stroke management, there was a lack of emphasis on targeted preventive measures for pneumonia. This study evaluates the impact of airway management guided by Protection Motivation Theory (PMT) on preventing post-stroke pneumonia.

**Methods:**

A retrospective study was conducted with 100 stroke patients admitted to the general neurology ward between January and December 2023. Patients were divided into two groups based on chronological admission order: 50 received standard airway management (January–June 2023), and 50 received PMT-guided intervention (July–December 2023). The PMT group engaged in structured educational sessions (30 min daily for 7 days) and actionable coping strategies to enhance adherence to airway management. Outcomes assessed included incidence of post-stroke pneumonia (diagnosed by chest CT within 7 days post-admission), respiratory function, length of hospital stay, and cognitive and psychological measures.

**Results:**

The PMT group showed a lower incidence of pneumonia (16% vs. 34%, *p* = 0.038) and reduced hospital stay (13.47 ± 3.86 days vs. 15.72 ± 4.36 days, *p* = 0.007). The absolute risk reduction was 18% with a number needed to treat (NNT) of 5.6. Improvements were noted in respiratory function, with higher forced vital capacity (2.46 ± 0.68 L vs. 2.15 ± 0.56 L, *p* = 0.013). Cognitive function, as measured by the Montreal Cognitive Assessment, was enhanced (23.58 ± 4.06 vs. 21.35 ± 3.84, *p* = 0.006), with both groups remaining below the normal threshold of 26 points. Depression levels were reduced (PHQ-9: 12.05 ± 3.12 vs. 13.46 ± 3.56, *p* = 0.038).

**Conclusion:**

PMT-guided airway management significantly enhances post-stroke outcomes through improved respiratory function, reduced pneumonia incidence, and better cognitive and psychological wellbeing. Future prospective studies with larger sample sizes are warranted to validate these findings.

## 1 Introduction

Stroke remains a leading cause of morbidity and mortality globally, with a substantial number of patients experiencing complications that worsen their prognosis and quality of life (1). Among these complications, post-stroke pneumonia represents a significant clinical concern, contributing to extended hospital stays, increased healthcare costs, and raised mortality rates (2). The pathophysiology of post-stroke pneumonia was complex, involving impaired swallowing and respiratory function, reduced mobility, and altered immune responses (3). Despite advancements in stroke management, preventive strategies targeting these complications were urgently needed to improve patient outcomes (4).

Conventional management of stroke patients often focuses on medical interventions for thromboprophylaxis and rehabilitation exercises (5). However, less emphasis was placed on preventive measures tailored toward reducing the risk of pneumonia (6). Airway management was a critical component in this context, given the vulnerability to aspiration and the potential for respiratory dysfunction (7). Existing research highlights the importance of meticulous airway care, yet standardized approaches incorporating cognitive and behavioral theories have not been thoroughly investigated (8).

The Protection Motivation Theory (PMT), originally developed within the field of health psychology, offers a robust framework for motivating protective behaviors through cognitive appraisal processes (9). PMT posits that the intention to engage in protective actions was influenced by perceived severity and vulnerability to a health threat, alongside the perceived efficacy and self-efficacy of the preventive behavior (10). This cognitive model provides a structured approach for motivating patients and caregivers to adhere to medical guidelines, especially in chronic disease management (11).

In recent years, PMT has been increasingly applied in healthcare settings to enhance patient compliance and behavioral adherence (12). Its utility in airway management could be transformative, particularly in the context of stroke, where patient adherence to therapeutic regimes was often suboptimal due to cognitive impairment and reduced motivation (13). By integrating PMT into airway management protocols, there was potential to foster proactive health behaviors and mitigate the risk of post-stroke pneumonia (14).

This study explores the impact of airway management strategies guided by PMT principles on the prevention and prognosis of post-stroke pneumonia. Grounded in behavioral science, the intervention was designed to engage both patients and caregivers through structured educational sessions and actionable coping strategies.

## 2 Materials and methods

### 2.1 Study design

This retrospective study evaluates stroke patients admitted to the general neurology ward of our hospital between January 2023 and December 2023. Among these patients, 50 underwent standard airway management (admitted January–June 2023), while the other 50 received airway management guided by the principles of the PMT (admitted July–December 2023). Patients were enrolled within 48 h of stroke onset and observed for 7 days with a study endpoint at discharge or 30 days post-admission, whichever came first.

### 2.2 Ethics statement

This study was conducted in accordance with the Declaration of Helsinki and approved by the Ethics Committee of Nanjing Gaochun People’s Hospital (JS-NJ-010). Because the research relied solely on de-identified patient data and did not pose any risk or affect patient care, informed consent was not required for this retrospective study. This consent waiver was granted by the Institutional Review Board and Ethics Committee, adhering to the regulatory and ethical standards applicable to retrospective research.

### 2.3 Eligibility and grouping criteria

Inclusion criteria for the study were as follows: patients must meet the diagnostic criteria for stroke confirmed by MRI, CT, or other imaging modalities; be 18 years of age or older; voluntarily consent to participate in the study; possess a stable general condition and be conscious; have complete clinical data available; and be admitted to the general neurology ward within 48 h of stroke onset.

Exclusion criteria were as follows: patients with psychiatric or cognitive impairments; those with concurrent conditions such as intracranial tumors, meningiomas, head trauma, intracranial infections, shock, or a history of head surgery; patients with cardiac or renal insufficiency; those with local oral or pharyngeal lesions; and patients requiring immediate ICU admission.

### 2.4 Post-hoc power analysis

Prior to data analysis, a post-hoc power analysis was conducted using G*Power 3.1.9.7 for t-tests under the “Means: Difference between two independent means (two groups)” option. The settings included a two-tailed test with an effect size (d) of 0.6 and an alpha error probability (*α*) of 0.05. After inputting the sample sizes for the two groups (*n* = 50 per group), the calculated power (1-β error probability) was determined to be 0.844, indicating adequate statistical power for detecting meaningful differences between groups.

### 2.5 Treatment approach

**Control group (standard airway management):** The control group received comprehensive standard care including: (1) dietary guidance with texture modification based on dysphagia severity; (2) positioning with 30-degree head-of-bed elevation; (3) oral hygiene performed twice daily using chlorhexidine solution; (4) regular suctioning every 4–6 h or as needed; (5) chest physiotherapy including percussion and vibration; (6) repositioning every 2–3 h to alternate sides for bedridden patients; and (7) continuous monitoring of vital signs and oxygen saturation.

**PMT group (PMT-guided airway management):** Prior to intervention, a multidisciplinary team was formed comprising at least two attending physicians and three nurses trained in PMT principles. The PMT intervention included all standard care components plus:

1 **Structured educational sessions**: Daily 30-min sessions for 7 days covering: threat appraisal (understanding pneumonia risks), coping appraisal (learning preventive strategies), self-efficacy building (practicing airway clearance techniques), and response efficacy (understanding intervention benefits).
2 **Enhanced monitoring protocol**: Oral cavity assessment every 2–4 h; tracheal humidity monitoring with pH detection every 6–12 h; increased inspection frequency to every 1–3 h for fixation verification.
3 **Family engagement program**: Training family members in basic respiratory assessment, mucus evaluation, and documentation using structured forms; provision of bedside educational materials and emergency contact protocols.
4 **Individualized risk assessment**: Comprehensive evaluation considering obesity (BMI > 30), existing pressure ulcers, frequent suctioning needs (>6 times/day), and other risk factors with tailored intervention protocols.
5 **Quality improvement activities**: Weekly team meetings to review adverse events, protocol adherence, and continuous skill enhancement through case discussions.

### 2.6 Diagnostic criteria for post-stroke pneumonia

Post-stroke pneumonia was diagnosed based on the following criteria within 7 days of admission: (1) new or progressive infiltrate on chest CT scan performed at clinical suspicion; (2) at least two of the following: fever >38 °C, leukocytosis >12,000/μL or leukopenia <4,000/μL, purulent sputum; (3) onset >48 h after stroke admission. Chest CT was typically performed within 3–5 days post-stroke or earlier if clinical symptoms developed.

### 2.7 General information

Demographic and disease-related characteristics of the patients were extracted from the medical records system. These included age, gender, body mass index (BMI), diabetes status, hypertension, smoking history, education level, marital status, employment status, stroke type and severity, level of consciousness, history of previous stroke, atrial fibrillation, hyperlipidemia, chronic kidney disease, coronary artery disease, antithrombotic treatment, dysphagia, and chronic obstructive pulmonary disease.

Statistical analyses were conducted on various treatment-related factors, including the average length of hospital stay, antibiotic usage, duration of intubation and oxygen therapy, incidence and onset time of pneumonia, pneumonia severity, rate of mechanical ventilation, duration of ICU stay, and occurrence of postoperative complications.

### 2.8 Pulmonary function

Pulmonary function and respiratory muscle strength were assessed post-care using a spirometer (Master Screen, CareFusion, Germany). During pulmonary function testing, patients stood upright with their heads in a neutral position, wearing a nose clip and holding the mouthpiece securely. The recorded parameters included forced expiratory volume in one second (FEV1), forced vital capacity (FVC), the ratio of FEV1 to FVC (FEV1/FVC), and peak expiratory flow rate.

For assessing respiratory muscle strength, patients were seated upright. They performed a maximal inspiration immediately following a maximal expiration, maintaining an uninterrupted effort, and then exhaled slowly. This process was repeated three times with a 10-s interval between attempts, and the highest value was recorded as the maximal inspiratory pressure (MIP). After a 10-min rest, patients quickly performed a maximal exhalation following a maximal inhalation, repeating this three times. The highest value obtained was recorded as the maximal expiratory pressure (MEP).

### 2.9 Assessment of stroke severity and level of consciousness

The National Institutes of Health Stroke Scale (NIHSS) was used to assess neurological impairment, with scores ranging from 0 to 42. Higher scores denote greater impairment. Specifically, a score of 0–1 suggests normal or near-normal status; 1–4 indicates a mild stroke or small stroke; 5–15 suggests a moderate stroke; 15–20 denotes a moderately severe stroke; and 21–42 reflects a severe stroke. The NIHSS has demonstrated a Cronbach’s alpha coefficient of 0.689, indicating acceptable internal consistency (15).

The level of consciousness for both groups was assessed using the Glasgow Coma Scale (GCS), which has a total score of 15. Higher scores indicate greater alertness. The GCS has a Cronbach’s alpha of 0.78, demonstrating good internal consistency (16).

### 2.10 Functional independent measurement (FIM)

The FIM was widely used to evaluate post-stroke participation. It encompasses six domains of daily function: self-care, sphincter control, transfer, locomotion, communication, and social cognition. The FIM consists of 18 items, each scored based on the level of assistance required, from 1 (complete dependence) to 7 (complete independence). The total score ranges from 18 to 126. The FIM demonstrates high internal consistency, with a Cronbach’s alpha of 0.973 (17).

### 2.11 Barthel Index (BI)

The BI was an ordinal scale designed to assess performance in activities of daily living. Total scores range from 0, indicating total dependence, to 100, indicating total independence. Specifically, scores between 0 and 20 reflect complete dependence, 21–60 indicate severe dependence, 61–90 suggest moderate dependence, and 91–99 denote mild dependence. The scale has a Cronbach’s alpha of 0.81, indicating good internal consistency (18).

### 2.12 State–trait anxiety inventory (STAI)

The STAI comprises 40 items, divided into the State Anxiety Scale (S-AI) and the Trait Anxiety Scale (T-AI). Each item was rated on a 1–4 scale, with reverse scoring applied to positively worded items. Both the S-AI and T-AI have maximum scores of 80, with higher scores reflecting more severe anxiety levels in patients. The scale’s Cronbach’s alpha was 0.842, indicating high reliability (19).

### 2.13 Patient health Questionnaire-9 (PHQ-9)

The PHQ-9 consists of 9 items, each rated on a 4-point scale from 0 to 3, resulting in a total score ranging up to 27. Higher scores indicate more severe levels of depression. The scale has a Cronbach’s alpha of 0.78, demonstrating good reliability (20).

### 2.14 Montreal cognitive assessment (MoCA)

The MoCA scale comprises 11 items across eight cognitive domains: attention and concentration, executive functions, memory, language, visuospatial skills, abstract thinking, calculation, and orientation. The total score was 30 points, with scores of 26 or above signifying normal cognitive function. Scores between 21 and 25 suggest moderate cognitive impairment, while scores from 0 to 20 indicate severe cognitive impairment. The MoCA scale has a Cronbach’s alpha coefficient of 0.87, reflecting strong internal consistency (21).

### 2.15 36-item short form health survey (SF-36)

The SF-36 was a health survey instrument divided into eight dimensions: physical functioning (PF), role limitations due to physical health (RP), bodily pain (BP), general health perceptions (GH), vitality (VT), social functioning (SF), role limitations due to emotional problems (RE), and mental health (MH). Each dimension was scored out of 100 points, with higher scores signifying better quality of life in that specific area. The SF-36 has a Cronbach’s alpha coefficient of 0.87, indicating strong internal consistency (22).

### 2.16 Statistical analysis

The data were analyzed using SPSS 29.0 statistical software (SPSS Inc., Chicago, IL, USA). Categorical data were represented as [*n* (%)]. The chi-square test was applied in its standard form when the sample size was ≥40 and the theoretical frequency (T) was ≥5, with the test statistic denoted by *χ*^2^. If the sample size was ≥40 but the theoretical frequency was between 1 ≤ *T* < 5, the chi-square test was adjusted using a correction formula. For sample sizes <40 or theoretical frequencies *T* < 1, Fisher’s exact probability method was utilized for statistical analysis.

Continuous variables were tested for normal distribution using the Shapiro–Wilk method. For normally distributed data, results were presented in the format (X ± s). Non-normally distributed data were analyzed using the Wilcoxon rank-sum test, and results were expressed as the [median (25% quantile, 75% quantile)]. A *p*-value of less than 0.05 was considered statistically significant.

## 3 Results

### 3.1 Demographic and basic data

In this study examining the impact of airway management guided by PMT on post-stroke pneumonia, we analyzed the baseline demographic characteristics of the participants across the conventional group and the PMT group, each consisting of 50 individuals (Table 1). The mean age of participants was similar between the groups, with the conventional group at 68.24 ± 7.54 years and the PMT group at 67.43 ± 8.13 years (*p* = 0.607). Gender distribution was also comparable, with 50.00% males in the conventional group and 56.00% males in the PMT group (*p* = 0.548). BMI variations were minor between groups (*p* = 0.777). The prevalence of hypertension (*p* = 0.673) and diabetes mellitus (*p* = 0.648) showed no statistically significant differences. Smoking status was nearly identical with 28.00% in the conventional group compared to 26.00% in the PMT group (*p* = 0.822). Additionally, no significant differences were found concerning education level, marital status, or employment status (*p* > 0.05). These results suggest homogeneity in demographic characteristics across the study groups, indicating a balanced allocation of participants.

**Table 1:** Demographic characteristics of the study population.

Characteristic	Conventional group (*n* = 50)	PMT group (*n* = 50)	*t*/*χ*^2^	*p*
Age (years)	68.24 ± 7.54	67.43 ± 8.13	0.516	0.607
Gender (male, %)	25 (50.00%)	28 (56.00%)	0.361	0.548
BMI (kg/m^2^)	26.73 ± 3.48	26.54 ± 3.26	0.283	0.777
Hypertension (%)	34 (68.00%)	32 (64.00%)	0.178	0.673
Diabetes mellitus (%)	14 (28.00%)	12 (24.00%)	0.208	0.648
Smoking status (%)	14 (28.00%)	13 (26.00%)	0.051	0.822
Education level (%)			0.885	0.829
No formal education	2 (4.00%)	4 (8.00%)		
High school	20 (40.00%)	18 (36.00%)		
College/University	23 (46.00%)	22 (44.00%)		
Postgraduate	5 (10.00%)	6 (12.00%)		
Marital status (%)			0.672	0.714
Single	11 (22.00%)	8 (16.00%)		
Married	29 (58.00%)	30 (60.00%)		
Widowed/divorced	10 (20.00%)	12 (24.00%)		
Employment status (%)			0.727	0.695
Employed	18 (36.00%)	20 (40.00%)		
Retired	23 (46.00%)	24 (48.00%)		
Unemployed	9 (18.00%)	6 (12.00%)		

### 3.2 Baseline disease-related features

The distribution of stroke types was similar, with 82% ischemic and 18% hemorrhagic in the conventional group, compared to 80% ischemic and 20% hemorrhagic in the PMT group (*p* = 0.799) (Table 2). Stroke severity, as measured by the NIHSS, averaged 7.52 ± 2.35 in the conventional group and 7.35 ± 2.14 in the PMT group (*p* = 0.720). Consciousness levels were assessed using the GCS, with scores of 11.24 ± 1.87 and 11.64 ± 1.54 for the conventional and PMT groups, respectively (*p* = 0.251). Additionally, previous stroke history, atrial fibrillation, hyperlipidemia, chronic kidney disease, coronary artery disease, and antithrombotic therapy usage were comparable between the groups. Dysphagia was present in 42% of the conventional group and 36% of the PMT group (*p* = 0.539), while chronic obstructive pulmonary disease was noted in 12 and 16% of participants, respectively (*p* = 0.564). These findings indicate that the two groups were well-matched with respect to baseline disease-related characteristics.

**Table 2:** Baseline disease-related features of the study population.

Feature	Conventional group (*n* = 50)	PMT group (*n* = 50)	*t*/*χ*^2^	*p*
Type of stroke (%)			0.065	0.799
Ischemic	41 (82.00%)	40 (80.00%)		
Hemorrhagic	9 (18.00%)	10 (20.00%)		
Stroke severity (NIHSS)	7.52 ± 2.35	7.35 ± 2.14	0.359	0.720
Consciousness level (GCS)	11.24 ± 1.87	11.64 ± 1.54	1.154	0.251
Previous stroke history (%)	18 (36.00%)	17 (34.00%)	0.044	0.834
Atrial fibrillation (%)	13 (26.00%)	11 (22.00%)	0.219	0.640
Hyperlipidemia (%)	29 (58.00%)	32 (64.00%)	0.378	0.539
Chronic kidney disease (%)	8 (16.00%)	9 (18.00%)	0.071	0.790
Coronary artery disease (%)	16 (32.00%)	17 (34.00%)	0.045	0.832
Antithrombotic therapy usage (%)	31 (62.00%)	29 (58.00%)	0.167	0.683
Dysphagia present (%)	21 (42.00%)	18 (36.00%)	0.378	0.539
Chronic obstructive pulmonary disease (COPD) (%)	6 (12.00%)	8 (16.00%)	0.332	0.564

### 3.3 Respiratory function and rehabilitation

The PMT group demonstrated a higher FVC of 2.46 ± 0.68 L compared to 2.15 ± 0.56 L in the conventional group (*t* = 2.530, *p* = 0.013) (Table 3). Additionally, peak expiratory flow rate was significantly greater in the PMT group at 380.47 ± 50.26 L/min, versus 350.47 ± 45.37 L/min in the conventional group (*t* = 3.133, *p* = 0.002). Improvements were also noted in inspiratory muscle strength, with the PMT group achieving 50.62 ± 9.15 cmH2O compared to 45.26 ± 8.12 cmH2O in the conventional group (*t* = 3.096, *p* = 0.003), and in expiratory muscle strength, where the PMT group recorded 67.29 ± 5.75 cmH2O against 64.14 ± 5.48 cmH2O in the conventional group (*t* = 2.802, *p* = 0.006). There was no statistically significant difference in the FEV1/FVC ratio between the groups (*t* = 0.959, *p* = 0.340). Overall, the PMT-guided intervention was associated with improved respiratory function parameters.

**Table 3:** Respiratory function and rehabilitation.

Respiratory parameter	Conventional group (*n* = 50)	PMT group (*n* = 50)	*t*	*p*
Forced vital capacity (FVC, L)	2.15 ± 0.56	2.46 ± 0.68	2.530	0.013
FEV1/FVC ratio	80.26 ± 5.14	81.16 ± 4.15	0.959	0.340
Peak expiratory flow rate (L/min)	350.47 ± 45.37	380.47 ± 50.26	3.133	0.002
Inspiratory muscle strength (cmH_2_O)	45.26 ± 8.12	50.62 ± 9.15	3.096	0.003
Expiratory muscle strength (cmH_2_O)	64.14 ± 5.48	67.29 ± 5.75	2.802	0.006

### 3.4 Treatment related variables

The PMT group exhibited a shorter average hospital stay of 13.47 ± 3.86 days compared to 15.72 ± 4.36 days in the conventional group (*t* = 2.734, *p* = 0.007) (Table 4). Additionally, the duration of oxygen therapy was significantly reduced in the PMT group, averaging 8.96 ± 1.96 days as opposed to 10.28 ± 2.15 days in the conventional group (*t* = 3.212, *p* = 0.002). The necessity for intubation was markedly lower in the PMT group, with only 10.00% requiring intubation compared to 26.00% in the conventional group (*χ*^2^ = 4.336, *p* = 0.037). There were no statistically significant differences in the rates of antibiotic use between the groups, with 80.00% in the conventional group and 70.00% in the PMT group utilizing antibiotics (*χ*^2^ = 1.333, *p* = 0.248). These findings suggest that the PMT-guided intervention may lead to improved treatment outcomes, characterized by reduced hospital stay, decreased duration of oxygen therapy, and less need for intubation.

**Table 4:** Treatment related variables.

Variable	Conventional group (*n* = 50)	PMT group (*n* = 50)	*t*/*χ*^2^	*p*
Average hospital stay (days)	15.72 ± 4.36	13.47 ± 3.86	2.734	0.007
Use of antibiotics (%)	40 (80.00%)	35 (70.00%)	1.333	0.248
Intubation necessity (%)	13 (26.00%)	5 (10.00%)	4.336	0.037
Oxygen therapy duration (days)	10.28 ± 2.15	8.96 ± 1.96	3.212	0.002

### 3.5 Incidence of post-stroke pneumonia

The incidence of pneumonia was significantly lower in the PMT group at 16.00% compared to 34.00% in the conventional group (*χ*^2^ = 4.320, *p* = 0.038) (Table 5). This represents an absolute risk reduction of 18% and a number needed to treat (NNT) of 5.6, indicating that approximately 6 patients need to be treated with PMT-guided management to prevent one case of post-stroke pneumonia. The average time to pneumonia onset was also reduced in the PMT group, recorded at 4.32 ± 1.24 days, as opposed to 5.21 ± 1.56 days in the conventional group (*t* = 3.176, *p* = 0.002). Pneumonia severity distribution favored the PMT group, with fewer severe cases (18.00% mild, 58.00% moderate, and 24.00% severe in the conventional group versus 38.00% mild, 52.00% moderate, and 10.00% severe in the PMT group; *χ*^2^ = 6.617, *p* = 0.037). Additionally, the need for mechanical ventilation was significantly lower in the PMT group, at 8.00% compared with 24.00% in the conventional group (*χ*^2^ = 4.762, *p* = 0.029). The length of ICU stay was also shorter in the PMT group, averaging 5.31 ± 1.83 days versus 6.35 ± 2.54 days in the conventional group (*t* = 2.348, *p* = 0.021). These results indicate that PMT-guided management significantly reduces the incidence, early onset, and severity of post-stroke pneumonia, and decreases the need for mechanical ventilation and ICU stay duration.

**Table 5:** Incidence of post-stroke pneumonia.

Outcome	Conventional group (*n* = 50)	PMT group (*n* = 50)	*t*/*χ*^2^	*p*
Pneumonia incidence (%)	17 (34.00%)	8 (16.00%)	4.320	0.038
Time to onset (days)	5.21 ± 1.56	4.32 ± 1.24	3.176	0.002
Severity (mild/moderate/severe)	9 (18.00.%)/29 (58.00%)/12 (24.00%)	19 (38.00%)/26 (52.00%)/5 (10.00%)	6.617	0.037
Mechanical ventilation (%)	12 (24.00%)	4 (8.00%)	4.762	0.029
Length of ICU stay (days)	6.35 ± 2.54	5.31 ± 1.83	2.348	0.021

### 3.6 Functional outcomes after treatment

**Figure 1:**
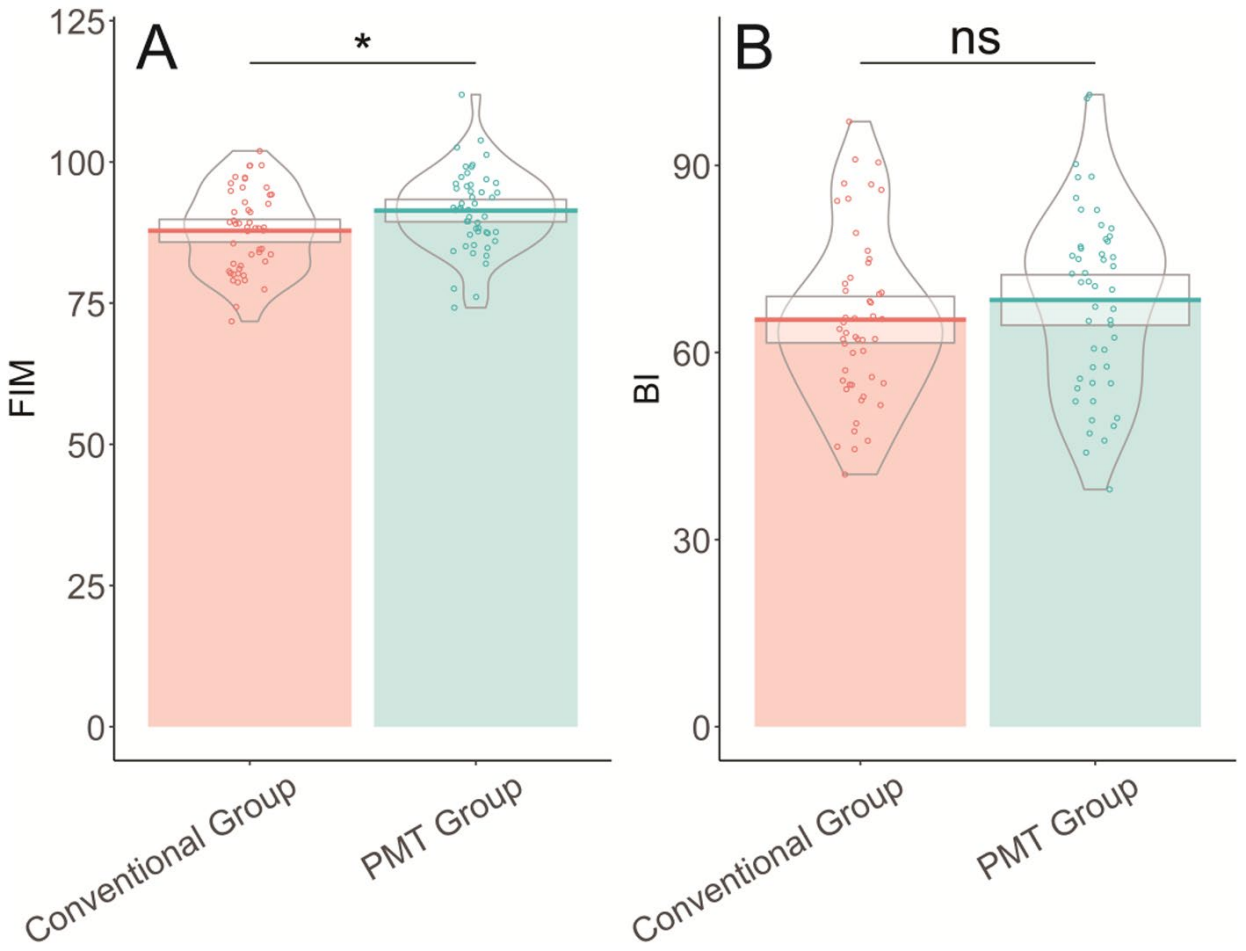
Functional outcomes after treatment. **(A)** Shows the Functional Independence Measurement (FIM) scores comparing conventional group (mean 87.82 ± 7.25) versus PMT group (mean 91.38 ± 7.12), demonstrating significantly higher functional independence in the PMT group (*p* = 0.015). **(B)** Displays the Barthel Index (BI) scores with no significant difference between conventional group (mean 65.26 ± 13.42) and PMT group (mean 68.42 ± 14.57) (*p* = 0.263).

The analysis of functional outcomes following treatment revealed that the PMT group had a significantly higher Functional Independence Measurement (FIM) score, averaging 91.38 ± 7.12, compared to 87.82 ± 7.25 in the conventional group (*t* = 2.477, *p* = 0.015), indicating greater independence in daily activities (Table 6). The Barthel Index (BI) scores showed no statistically significant difference between the groups, with the PMT group scoring 68.42 ± 14.57 and the conventional group scoring 65.26 ± 13.42 (*t* = 1.126, *p* = 0.263). These results suggest that airway management guided by PMT positively influences functional independence post-treatment, although the overall daily living abilities, as measured by the Barthel Index, did not differ significantly (Figure 1).

**Table 6:** Cognitive outcomes.

Cognitive measure	Conventional group (*n* = 50)	PMT group (*n* = 50)	*t*	*p*
Cognitive assessment (MoCA)	21.35 ± 3.84	23.58 ± 4.06	2.823	0.006
Memory recall test (score)	7.63 ± 1.65	8.41 ± 1.58	2.424	0.017
Attention span (score)	6.54 ± 1.43	7.36 ± 1.24	3.058	0.003
Processing speed (sec)	39.23 ± 4.58	38.16 ± 4.05	1.239	0.218
Executive function (score)	8.67 ± 1.96	9.62 ± 1.83	2.512	0.014

### 3.7 Psychological assessment

**Figure 2:**
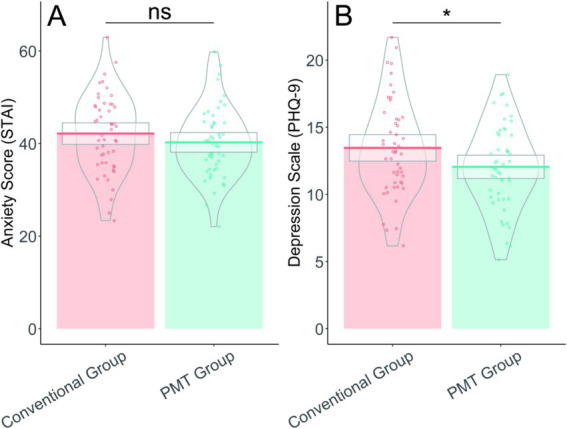
Psychological assessment. **(A)** Illustrates the State–Trait Anxiety Inventory (STAI) scores showing no significant difference between conventional group (mean 42.15 ± 8.38) and PMT group (mean 40.26 ± 7.65) (*p* = 0.240). **(B)** Demonstrates the Patient Health Questionnaire-9 (PHQ-9) depression scores with significantly lower values in the PMT group (mean 12.05 ± 3.12) compared to conventional group (mean 13.46 ± 3.56) (*p* = 0.038).

Psychological assessments post-treatment indicated that the PMT group experienced a significantly lower level of depression, with a PHQ-9 score of 12.05 ± 3.12 compared to 13.46 ± 3.56 in the conventional group (*t* = 2.103, *p* = 0.038), suggesting a beneficial impact of the intervention on depressive symptoms (Table 7). However, there was no statistically significant difference in anxiety levels between the groups; the STAI score was 40.26 ± 7.65 in the PMT group and 42.15 ± 8.38 in the conventional group (*t* = 1.181, *p* = 0.240). These results imply that while the PMT-guided airway management approach can reduce depression, it does not significantly affect anxiety levels in post-stroke patients (Figure 2).

**Table 7:** Health-related quality of life (SF-36).

Quality of life parameter	Conventional group (*n* = 50)	PMT group (*n* = 50)	*t*	*p*
Physical functioning (score)	45.62 ± 8.91	50.24 ± 7.82	2.753	0.007
Mental health (score)	42.35 ± 9.12	46.83 ± 8.51	2.542	0.013
Social functioning (score)	48.05 ± 7.24	51.52 ± 7.03	2.434	0.017
Bodily pain (score)	5.87 ± 2.16	4.98 ± 1.85	2.223	0.029
Vitality (score)	40.16 ± 7.65	44.76 ± 7.31	3.077	0.003

### 3.8 Cognitive outcomes

The MoCA score was higher in the PMT group, averaging 23.58 ± 4.06 compared to 21.35 ± 3.84 in the conventional group (*t* = 2.823, *p* = 0.006), demonstrating enhanced overall cognitive function (Table 6). While this difference is statistically significant, it should be noted that both groups remained below the normal cognitive threshold of 26 points, indicating persistent moderate cognitive impairment. Memory recall improved markedly in the PMT group, with a score of 8.41 ± 1.58 versus 7.63 ± 1.65 in the conventional group (*t* = 2.424, *p* = 0.017). Additionally, the PMT group showed superior attention span scores (7.36 ± 1.24) compared to the conventional group (6.54 ± 1.43; *t* = 3.058, *p* = 0.003) and better performance in executive function (9.62 ± 1.83 versus 8.67 ± 1.96; *t* = 2.512, *p* = 0.014). However, no statistically significant difference was observed in processing speed, with the PMT group recording 38.16 ± 4.05 s and the conventional group 39.23 ± 4.58 s (*t* = 1.239, *p* = 0.218). These findings suggest that airway management guided by PMT significantly enhances cognitive function in areas such as general cognition, memory, attention, and executive function in post-stroke patients.

### 3.9 Health-related quality of life (SF-36)

PF scores were notably higher in the PMT group, averaging 50.24 ± 7.82 compared to 45.62 ± 8.91 in the conventional group (*t* = 2.753, *p* = 0.007) (Table 7). MH was also enhanced, with PMT participants scoring 46.83 ± 8.51 versus 42.35 ± 9.12 in the conventional group (*t* = 2.542, *p* = 0.013). SF improved significantly in the PMT group, with scores of 51.52 ± 7.03 compared to 48.05 ± 7.24 in the conventional group (*t* = 2.434, *p* = 0.017). Additionally, BP scores were lower in the PMT group, indicating less perceived pain (4.98 ± 1.85 vs. 5.87 ± 2.16; *t* = 2.223, *p* = 0.029). VT scores were higher as well, with the PMT group achieving 44.76 ± 7.31 compared to 40.16 ± 7.65 in the conventional group (*t* = 3.077, *p* = 0.003). These findings suggest that airway management guided by PMT significantly enhances several aspects of health-related quality of life in post-stroke patients.

### 3.10 Complications related to airway management

The incidence of aspiration events was reduced to 4.00% in the PMT group, compared to 18.00% in the conventional group (*χ*^2^ = 5.005, *p* = 0.025) (Table 8). Hypoxia occurred less frequently in the PMT group, with an incidence of 2.00% compared to 16.00% in the conventional group (*χ*^2^ = 4.396, *p* = 0.036). Similarly, infection at the insertion site was significantly lower in the PMT group at 2.00%, compared to 16.00% in the conventional group (*χ*^2^ = 4.396, *p* = 0.036). There were no statistically significant differences in the rates of tracheal injury (*p* = 0.610) or intubation difficulty (*p* = 0.505) between the groups. These results suggest that airway management guided by PMT effectively reduces the risk of certain complications associated with airway management in post-stroke patients.

**Table 8:** Complications related to airway management.

Complication	Conventional group (*n* = 50)	PMT group (*n* = 50)	*χ* ^2^	*p*
Aspiration event (%)	9 (18.00%)	2 (4.00%)	5.005	0.025
Hypoxia incidence (%)	8 (16.00%)	1 (2.00%)	4.396	0.036
Tracheal injury (%)	3 (6.00%)	1 (2.00%)	0.260	0.610
Infection at insertion site (%)	8 (16.00%)	1 (2.00%)	4.396	0.036
Intubation difficulty (%)	6 (12.00%)	4 (8.00%)	0.444	0.505

## 4 Discussion

Central to the improved outcomes was the PMT approach’s emphasis on enhancing both patient and caregiver engagement through structured education and empowerment. By focusing on the principles of threat appraisal and coping strategies inherent in PMT, the intervention effectively motivated patients and their families to adhere to airway management practices. This motivation was possibly key to the reduction in pneumonia incidences. Enhanced vigilance toward potential complications like extubation or airway obstruction fosters an environment of proactive healthcare, thus mitigating risks associated with pneumonia and mechanical ventilation.

The incorporation of PMT appears to significantly impact respiratory function, as evidenced by improvements in forced vital capacity, peak expiratory flow rate, and respiratory muscle strength. These enhancements could be attributed to the increased frequency of monitoring and the participatory role of family members in the intervention group (23). Encouraging active involvement in care routines, including repositioning and percussion, likely leads to improved secretion clearance and lung expansion (24). Additionally, the implementation of structured health education sessions enhances understanding of the pathophysiological implications of airway management, motivating adherence and potentially resulting in improved compliance with airway exercises (25).

The PMT framework establishes clear linkages between cognitive appraisal processes and physiological outcomes through several mechanisms. First, threat appraisal enhances patients’ understanding of pneumonia severity, leading to increased vigilance in performing preventive behaviors. Second, self-efficacy building through hands-on training enables patients to effectively perform airway clearance techniques, directly improving respiratory parameters. Third, response efficacy education helps patients understand how specific interventions (such as positioning and oral hygiene) directly prevent aspiration, thereby increasing adherence (26). These cognitive-behavioral changes translate into measurable physiological improvements through consistent practice of protective behaviors and early intervention when complications arise (27).

Furthermore, the structured PMT intervention resulted in significantly shorter hospital stays and a reduced need for intubation and oxygen therapy (28). The cognitive framework based on PMT likely drives more diligent adherence to care protocols, minimizing the occurrence and severity of conditions that necessitate such interventions (29). This framework promotes anticipatory guidance and decision-making that aids in promptly addressing adverse symptoms or conditions.

The reduced incidence and severity of post-stroke pneumonia in the PMT group can be partially explained by the intervention’s tailored approach that factors in individual patient risks, such as obesity or existing ulcers, and adjusts management strategies accordingly. The meticulous assessment of each patient’s condition allows for timely interventions that address specific vulnerabilities, thereby helping to preempt the development of pneumonia (30). The PMT approach also likely fosters a more robust immune response by minimizing stress and promoting effective coping mechanisms, which were known to inversely impact susceptibility to infections (31).

Cognitive and psychological improvements observed in the PMT group reflect the intervention’s holistic approach, integrating cognitive-behavioral strategies with practical airway management techniques. The active involvement of patients in their care plan through self-scoring systems and regular discussions enhances cognitive engagement, which might correlate with improved cognitive outcomes like memory recall and executive function (32). The reduction in PHQ-9 scores in the PMT group suggests that empowerment through education and active participation has a positive impact on MH, potentially through increased self-efficacy and reduced anxiety about the disease process (33). While the reduction in depression scores was statistically significant, it should be noted that this improvement may be multifactorial, influenced not only by the PMT intervention but also by factors such as reduced hospital stay and improved functional outcomes (34).

The results indicate that PMT-guided interventions not only reduce the physical complications associated with post-stroke management but also promote enhanced psychological wellbeing and cognitive recovery. Education and social support systems embedded in PMT could lead to patients experiencing reduced feelings of helplessness, which were known to exacerbate depression and anxiety in stroke patients (35). Thus, the psychological and cognitive benefits likely stem from the comprehensive engagement encouraged by PMT.

Comparing our findings with existing literature, several studies have demonstrated the efficacy of structured interventions in reducing post-stroke pneumonia. A recent systematic review by Chen et al. reported that multimodal interventions incorporating dysphagia screening and oral care reduced pneumonia incidence by 15–20%, similar to our 18% absolute risk reduction (36). However, our study is among the first to apply PMT specifically to airway management in stroke patients. Previous applications of PMT in chronic disease management, such as diabetes and hypertension, have shown improvements in medication adherence and self-care behaviors, with effect sizes ranging from 0.4 to 0.7 (37). Our findings extend this evidence to acute stroke care, demonstrating that theory-based interventions can effectively reduce complications even in cognitively impaired populations (38).

Notably, the study highlights an intriguing interplay between educational interventions and physiological outcomes. By promoting understanding and anticipation of potential complications, the PMT framework likely improves adherence to medical advice, enhancing overall patient outcomes (39). Family involvement, encouraged by the PMT intervention, acts as a supplementary system of care, offering emotional support and reinforcing adherence to prescribed health behaviors (40). This supplementary support structure can be crucial in maintaining motivation and consistent care practices, leading to reduced respiratory and functional complications.

An area of discussion centers on the potential scalability and generalizability of PMT-guided management. The structured nature and the ability to tailor interventions based on patient-specific factors suggest that such an approach could be effectively adapted across diverse clinical settings and patient populations. However, further research focusing on long-term outcomes, cost-effectiveness, and implementation barriers was necessary to delineate the broader applicability of PMT in clinical practice.

While this study provides valuable insights into the impact of PMT-guided airway management on post-stroke pneumonia outcomes, there were several limitations that must be acknowledged. Firstly, the study’s sample size, though adequate for preliminary analysis based on our post-hoc power calculation (power = 0.844), may limit the generalizability of the findings across larger and more diverse populations. Additionally, the retrospective design introduces potential selection bias, as patients were grouped based on chronological admission periods rather than randomization. The comprehensive data collection in our study, while unusual for retrospective research, was facilitated by our institution’s standardized stroke care protocols and electronic medical records system. Nevertheless, the study’s design, relying largely on short-term follow-up (30 days maximum), does not capture long-term effects and sustainability of adherence to the PMT-guided protocols. The potential influence of unmeasured confounding variables, such as varying levels of caregiver support and differences in healthcare access, may also have impacted the outcomes. Furthermore, the reliance on self-reported adherence and psychological measures introduces the risk of response bias. The lack of detailed prospective sample size calculation, though addressed through post-hoc analysis, represents a methodological limitation. Future research should aim to address these limitations by employing larger, multi-center randomized controlled trials with extended follow-up periods and incorporating objective measures of adherence and psychological wellbeing to validate the robustness and applicability of the PMT framework in a broader clinical context.

## 5 Conclusion

In conclusion, the study underscores the critical impact of PMT-guided airway management on the prognosis of post-stroke pneumonia. The results highlight the importance of patient and caregiver involvement, facilitated through structured educational interventions and personalized care strategies. Through enhancing respiratory, psychological, and cognitive outcomes, the PMT framework offers a promising approach to managing post-stroke complications. The findings support the integration of behavioral theories into clinical protocols and warrant further investigation through prospective, randomized controlled trials to establish the long-term efficacy and cost-effectiveness of PMT-guided interventions in stroke care.

## Data Availability

The raw data supporting the conclusions of this article will be made available by the authors without undue reservation.

## References

[1] Wang L Wang Y Wang Y Wang F Zhang J Li S. Transcutaneous auricular vagus nerve stimulators: a review of past, present, and future devices. Expert Rev Med Devices. (2022) 19:43–61. doi: 10.1080/17434440.2022.2020095, PMID: 34937487

[2] Han YJ Jang YJ Park GY Joo YH Im S. Role of injection laryngoplasty in preventing post-stroke aspiration pneumonia, case series report. Medicine. (2020) 99:e19220. doi: 10.1097/MD.0000000000019220, PMID: 32049860 PMC7035062

[3] Sung KL Kuo MJ Hsieh CY Sung SF. High neutrophil-to-lymphocyte ratio predicts one-year risk of pneumonia post-stroke discharge. Cerebrovasc Dis (Basel, Switzerland). (2023) 52:567–74. doi: 10.1159/000529531, PMID: 36958294

[4] Sato S Yamana H Kumazawa R Watanabe H Fujita A Matsui H. Cilostazol versus aspirin or clopidogrel for reducing post-stroke aspiration pneumonia: a nationwide retrospective cohort study. Cerebrovasc Dis (Basel, Switzerland). (2024) 53:152–9. doi: 10.1159/000531716, PMID: 37586338 PMC10997247

[5] Kuo YW Huang YC Lee M Lee TH Lee JD. Risk stratification model for post-stroke pneumonia in patients with acute ischemic stroke. Eur J Cardiovasc Nurs. (2020) 19:513–20. doi: 10.1177/1474515119889770, PMID: 31735079

[6] Dziewas R Michou E Trapl-Grundschober M Lal A Arsava EM Bath PM. European Stroke Organisation and European Society for Swallowing Disorders guideline for the diagnosis and treatment of post-stroke dysphagia. Eur Stroke J. (2021) 6:Lxxxix-cxv. doi: 10.1177/23969873211039721, PMID: 34746431 PMC8564153

[7] Banda KJ Chu H Kang XL Liu D Pien LC Jen HJ. Prevalence of dysphagia and risk of pneumonia and mortality in acute stroke patients: a meta-analysis. BMC Geriatr. (2022) 22:420. doi: 10.1186/s12877-022-02960-5, PMID: 35562660 PMC9103417

[8] Xing D Chen YH Wang L Yu B Ran Z Chen L. Evaluation of the therapeutic effect of high-flow nasal cannula oxygen therapy on patients with aspiration pneumonia accompanied by respiratory failure in the post-stroke sequelae stage. BMC Pulm Med. (2021) 21:17. doi: 10.1186/s12890-020-01359-5, PMID: 33413281 PMC7788538

[9] Faura J Bustamante A Miró-Mur F Montaner J. Stroke-induced immunosuppression: implications for the prevention and prediction of post-stroke infections. J Neuroinflammation. (2021) 18:127. doi: 10.1186/s12974-021-02177-0, PMID: 34092245 PMC8183083

[10] Labeit B Jung A Ahring S Oelenberg S Muhle P Roderigo M. Relationship between post-stroke dysphagia and pharyngeal sensory impairment. Neurol Res Pract. (2023) 5:7. doi: 10.1186/s42466-023-00233-z, PMID: 36793109 PMC9933330

[11] Phan TG Beare R Bath PM Ievlieva S Ho S Ly J. Effect of alteplase, benzodiazepines and beta-blocker on post-stroke pneumonia: exploration of VISTA-acute. PLoS One. (2023) 18:e0281617. doi: 10.1371/journal.pone.0281617, PMID: 37126535 PMC10150972

[12] Xu Y Ge Y Zhou M Zhang Z. Clenbuterol, a selective β2-adrenergic receptor agonist, inhibits or limits post-stroke pneumonia, but increases infarct volume in MCAO mice. J Inflamm Res. (2022) 15:295–309. doi: 10.2147/JIR.S344521, PMID: 35058704 PMC8765548

[13] Leangpanich N Chuphanitsakun Y Pakaranodom K Kerdjarern K Poonual W. Scoring of post stroke pneumonia in Uttaradit hospital. J Multidiscip Healthc. (2019) 12:917–23. doi: 10.2147/JMDH.S218654, PMID: 31814729 PMC6863128

[14] Li C Ma M Dong S Hong Y Bao J Zhang Y. Statin treatment in the acute phase and the risk of post-stroke pneumonia: a retrospective cohort study. Front Neurol. (2021) 12:635079. doi: 10.3389/fneur.2021.635079, PMID: 34552547 PMC8450324

[15] Wiśniewski A Filipska K Puchowska M Piec K Jaskólski F Ślusarz R. Validation of a polish version of the national institutes of health stroke scale: do moderate psychometric properties affect its clinical utility? PLoS One. (2021) 16:e0249211. doi: 10.1371/journal.pone.0249211, PMID: 33798218 PMC8018641

[16] Caruana M Hackenbruch SN Grech V Farrugia R. Inconsistency in the application of Glasgow coma scale in pediatric patients. Med Princ Pract. (2024) 33:41–6. doi: 10.1159/000534797, PMID: 37899031 PMC10896613

[17] Colomer C Llorens R Sánchez C Ugart P Moliner B Navarro MD. Reliability and validity of the Spanish adaptation of the functional Independence measure + functional assessment measure. Eur J Phys Rehabil Med. (2023) 59:452–7. doi: 10.23736/S1973-9087.23.07841-3, PMID: 37226445 PMC10548398

[18] Dos Reis NF Figueiredo F Biscaro RRM Lunardelli EB Maurici R. Psychometric properties of the Barthel index used at intensive care unit discharge. Am J Crit Care. (2022) 31:65–72. doi: 10.4037/ajcc2022732 34972844

[19] Du Q Liu H Yang C Chen X Zhang X. The development of a short Chinese version of the state-trait anxiety inventory. Front Psych. (2022) 13:854547. doi: 10.3389/fpsyt.2022.854547, PMID: 35619610 PMC9128482

[20] Degefa M Dubale B Bayouh F Ayele B Zewde Y. Validation of the PHQ-9 depression scale in Ethiopian cancer patients attending the oncology clinic at Tikur Anbessa specialized hospital. BMC Psychiatry. (2020) 20:446. doi: 10.1186/s12888-020-02850-3, PMID: 32912183 PMC7488004

[21] Khatib N El Harch I Lamkaddem A Omari L Attiya N Filali-Zegzouti Y. The Moroccan MoCA test: translation, cultural adaptation, and validation. Appl Neuropsychol Adult. (2022) 31:1256–60. doi: 10.1080/23279095.2022.2119143, PMID: 36089915

[22] Wu Q Chen Y Zhou Y Zhang X Huang Y Liu R. Reliability, validity, and sensitivity of short-form 36 health survey (SF-36) in patients with sick sinus syndrome. Medicine. (2023) 102:e33979. doi: 10.1097/MD.0000000000033979, PMID: 37327281 PMC10270486

[23] Xu F Bai L Dai Z Cheng H. Research hotspots and trends in post-stroke dysphagia: a bibliometric analysis. Front Neurosci. (2023) 17:1275748. doi: 10.3389/fnins.2023.1275748, PMID: 37942140 PMC10628302

[24] Li X Yu J Shu C. Bibliometric analysis of global research trends on post-stroke pneumonia: current development status and research Frontiers. Front Public Health. (2022) 10:950859. doi: 10.3389/fpubh.2022.950859, PMID: 35983361 PMC9379091

[25] Jung M Park HY Park GY Lee JI Kim Y Kim YH. Post-stroke infections: insights from big data using clinical data warehouse (CDW). Antibiotics (Basel, Switzerland). (2023) 12:740. doi: 10.3390/antibiotics12040740, PMID: 37107102 PMC10134983

[26] Seedat J Penn C. Implementing oral care to reduce aspiration pneumonia amongst patients with dysphagia in a South African setting. S Afr J Commun Disord. (2016) 63:102. doi: 10.4102/sajcd.v63i1.102 26974243 PMC8631170

[27] Gotaas ME Stiles TC Bjørngaard JH Borchgrevink PC Fors EA. Cognitive Behavioral Therapy Improves Physical Function and Fatigue in Mild and Moderate Chronic Fatigue Syndrome: A Consecutive Randomized Controlled Trial of Standard and Short Interventions. Front Psychiatry. (2021) 12:580924. doi: 10.3389/fpsyt.2021.580924 33912079 PMC8071989

[28] Szylińska A Kotfis K Bott-Olejnik M Wańkowicz P Rotter I. Post-stroke outcomes of patients with chronic obstructive pulmonary disease. Brain Sci. (2022) 12:106. doi: 10.3390/brainsci12010106, PMID: 35053849 PMC8774103

[29] Park HY Oh HM Kim TW Kim Y Park GY Hwang H. Single nucleotide polymorphisms may increase the risk of aspiration pneumonia in post-stroke patients with dysphagia. Curr Issues Mol Biol. (2022) 44:3735–45. doi: 10.3390/cimb44080255, PMID: 36005151 PMC9406641

[30] Tashima H Ito M Kawakami M Ishii R Miyazaki Y Akimoto T. Risk factors for post-stroke pneumonia in a patient population with subacute stroke: a retrospective cohort study. J Clin Med. (2023) 12:5835. doi: 10.3390/jcm12185835, PMID: 37762776 PMC10532161

[31] Akimoto T Hara M Ishihara M Ogawa K Nakajima H. Post-stroke pneumonia in real-world practice: background, microbiological examination, and treatment. Neurol Int. (2023) 15:69–77. doi: 10.3390/neurolint15010006, PMID: 36648970 PMC9844281

[32] Nishimura T Matsugaki R Matsuda S. Physical rehabilitation and post-stroke pneumonia: a retrospective observational study using the Japanese diagnosis procedure combination database. Neurol Int. (2023) 15:1459–68. doi: 10.3390/neurolint15040094, PMID: 38132973 PMC10745980

[33] Jagdmann S Dames C Berchtold D Winek K Weitbrecht L Meisel A. Impact of key nicotinic AChR subunits on post-stroke pneumococcal pneumonia. Vaccine. (2020) 8:253. doi: 10.3390/vaccines8020253, PMID: 32481512 PMC7349987

[34] Gadelrab HF Cabello M Vieta E Valle J Leonardi M Chatterji S. Explaining functional outcomes in depression treatment: a multilevel modelling approach. Disabil Rehabil. (2010) 32:S105–15. doi: 10.3109/09638288.2010.520808 20874448

[35] Kaylor SA Singh SA. Clinical outcomes associated with speech, language and swallowing difficulties post-stroke. South African J Commun Disord = Die Suid-Afrikaanse tydskrif vir Kommunikasieafwykings. (2023) 70:e1–e15. doi: 10.4102/sajcd.v70i1.957, PMID: 37916686 PMC10623651

[36] Siao SF Ku SC Tseng WH Wei YC Chang YC Hsiao TY. Effects of a swallowing and oral-care program on resuming oral feeding and reducing pneumonia in patients following endotracheal extubation: a randomized, open-label, controlled trial. Crit Care. (2023) 27:283. doi: 10.1186/s13054-023-04568-6 37438759 PMC10339550

[37] Lee SK Shin DH Kim YH Lee KS. Effect of Diabetes Education Through Pattern Management on Self-Care and Self-Efficacy in Patients with Type 2 Diabetes. Int J Environ Res Public Health. (2019) 16:3323. doi: 10.3390/ijerph16183323 31505892 PMC6765832

[38] Han C Zhang L Liu J. Development and Reliability Testing of the Stroke Patient Protection Motivation Scale. Neuropsychiatr Dis Treat. (2022) 18:1341–1349. doi: 10.2147/NDT.S353111 35813611 PMC9270040

[39] Ke Z Liu W Chen FY Ge WY Li XP Fan XN. Intracerebral hemorrhage and absence of pneumonia are independent predictors for nasogastric tube removal of post-stroke dysphagia. Ann Indian Acad Neurol. (2023) 26:90–3. doi: 10.4103/aian.aian_809_22, PMID: 37034053 PMC10081559

[40] Rashid MH Kabir A Waris MU Salman U Zain S. Role of prophylactic antibiotics in critical care of stroke patients - a preventive approach to post-stroke infections? Cureus. (2020) 12:e7158. doi: 10.7759/cureus.7158, PMID: 32257701 PMC7108674

